# Textile Recycling: Efficient Polyester Recovery from Polycotton Blends Using the Heated High-Ethanol Alkaline Aqueous Process

**DOI:** 10.3390/polym16213008

**Published:** 2024-10-26

**Authors:** Kalliopi Elli Pavlopoulou, Kateřina Hrůzová, May Kahoush, Nawar Kadi, Alok Patel, Ulrika Rova, Leonidas Matsakas, Paul Christakopoulos

**Affiliations:** 1 Biochemical Process Engineering, Department of Civil, Environmental and Natural Resources Engineering, Luleå University of Technology, SE-971 87 Luleå, Sweden; kalliopi.elli.pavlopoulou@ltu.se (K.E.P.); katerina.hruzova@ltu.se (K.H.); alok.kumar.patel@ltu.se (A.P.); ulrika.rova@ltu.se (U.R.); 2 Department of Textile Technology, Faculty of Textiles, Engineering and Business, University of Borås, SE-501 90 Borås, Sweden; may.kahoush@hb.se (M.K.); nawar.kadi@hb.se (N.K.)

**Keywords:** polycotton, polyester, high-ethanol alkaline process, hydrolysis, textile recycling

## Abstract

Textile production has doubled in the last 20 years, but only 1% is recycled into new fibers. It is the third largest contributor to water pollution and land use, accounting for 10% of global carbon emissions and 20% of clean water pollution. A key challenge in textile recycling is blended yarns, such as polycotton blends, which consist of polyester and cotton. Chemical recycling offers a solution, in particular, alkali treatment, which hydrolyzes polyester (PET) into its components while preserving cotton fibers. However, conventional methods require high temperatures, long durations, or catalysts. Our study presents, for the first time, the heated high-ethanol alkaline aqueous (HHeAA) process that efficiently hydrolyzes PET from polycotton at lower temperatures and without a catalyst. A near-complete PET hydrolysis was achieved in 20 min at 90 °C, while similar results were obtained at 70 °C and 80 °C with longer reaction times. The process was successfully scaled at 90 °C for 20 min, and complete PET hydrolysis was achieved, with a significantly reduced liquid-to-solid ratio, from 40 to 7 (L per kg), signifying its potential to be implemented in an industrial context. Additionally, the cotton maintained most of its properties after the treatment. This method provides a more sustainable and efficient approach to polycotton recycling.

## 1. Introduction

In recent years, the fast fashion trend, fueled by social media and the faster pace of fashion trends, has led to a steep increase in the quantity of clothes that are produced and discarded. In 2000, global textile production was at 58 million tons, whereas in 2020, the production doubled to 109 million tons. The prediction for 2030 is 145 million tons of global textile production [1]. It is estimated that textile production is responsible for 10% of global carbon emissions and 20% of global clean water pollution, which made the textile industry the third largest source of water pollution and land use in 2020. With changing consumer behaviors, most clothes are being discarded instead of being donated; thus, about 11–15 kg of textiles per person are discarded in Europe annually [1,2]. According to Juanga-Labayen et al., globally, around 75% of textile waste is landfilled, highlighting the need for more advanced recycling technologies to address this growing problem [3]. The collection rate for textiles in Europe is around 30–35%; however, only 1% of used clothing was recycled into new fibers [1,4]. The critical aspect of recycling textiles lies in increasing collection rates, improving sorting efficiency, and developing energy-efficient and economically viable recycling technologies [2,5].

Commonly used textiles are mostly blends of multiple different fibers, which can be divided into natural and artificial [6]. Natural fibers include animal-based silk, wool, and hair (alpaca, cashmere, and angora), as well as plant-based cotton, jute, hemp, and many others. Artificial fibers can be further divided into natural and synthetic polymers. Natural polymers are made from rubber (elastodiene), regenerated protein (azlon), or regenerated cellulose (viscose, modal, and lyocell). Synthetic polymers are mostly fossil-based polyesters (PETs), polyamides (Nylon), and polyethers (Spandex) [6]. In 2022, cotton, polyester, and polyamide accounted for 22%, 54%, and 5% of global textile fiber production, with polycotton being the most prominent blend of cotton and polyesters [7].

Therefore, there is an urgent need for efficient recycling technologies that would manage the substantial volumes of these challenging wastes. Several methods have been developed in recent years, but the majority of technologies that are at a high technology readiness level (TRL) can only deal with a single-material fabric and also lead to the degradation of fiber quality [4,8,9]. These methods include mechanical and thermo-mechanical recycling, which yield new fabrics but cannot separate fiber blends due to their tight interweaving [4,5,10]. Thermochemical recycling transforms textiles into oils, gasses, char, aldehydes, and ketones through pyrolysis and gasification. Chemical recycling yields monomers of the polymers used in fabrics, and in some cases, such as PET, those monomers could be repolymerized again and new fibers could be produced [8,9,11,12]. Although only pure cotton textiles are currently recyclable, various applications for blends are being studied. However, the main drawbacks of the chemical methods are their generation of chemical waste and their water consumption. The combination of chemical recycling with circular economy principles, as discussed by Juanga-Labayen et al., is critical for reducing environmental burdens and creating sustainable textile recycling processes [3]. An emerging technology utilizing enzymes for textile recycling would also be able to yield monomers. However, more research must be conducted to make this method economically viable. Therefore, the challenge of the fiber blends cannot be currently tackled by one simple method [5,8].

**Figure 1:**
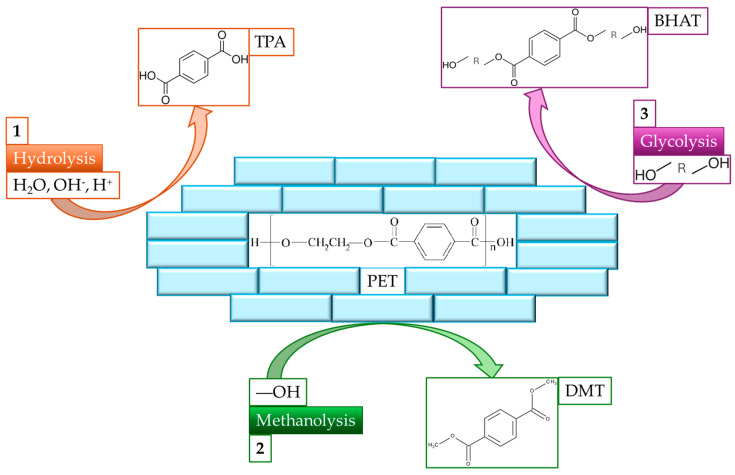
Illustration of the three main depolymerization reactions of PET and their main products. (1) Hydrolysis which produces terephthalic acid (TPA), (2) methanolysis which produces dimethyl terephthalate (DMT), and (3) glycolysis which produces bis(hydroxyalkyl)terephthalate (BHAT). All the reactions produce also ethylene glycol as a sub-product.

The prominence of polycotton blends calls for an efficient method for their recycling, and researchers have focused on developing chemical methods to achieve this goal [5]. Two main methods can be used: (1) chemical recycling of polymers, which uses solvents to dissolve cellulose or polyester fibers, and (2) chemical recycling of monomers, which breaks down polymers into monomers via hydrolysis or glycolysis [8]. The recycling of polymers requires high amounts of very specific and expensive solvents such as N-methylmorpholine N-oxide for cellulose or 1,3-dimethylimidazolidinone, butyl benzoate, and benzyl acetate for polyester. The inefficiencies of these processes, as highlighted by Damayanti et al., make them less attractive for large-scale applications, whereas monomer recycling offers a more sustainable route [13]. The dissolution of polymers often creates very viscous solutions; thus, high liquid-to-solid ratios must be employed, and this leads to a process that is not economically viable, even if a closed-loop system is considered. On the other hand, chemical recycling of monomers can be performed with a more common and cheaper set of chemicals, such as hydroxides, acids, methanol, or glycols [8,14]. Additionally, the recycling of monomers provides a solution to the quality deterioration issue arising from fiber recycling, as the monomers can be used to create virgin-quality fibers [11,12,15]. Currently, three main routes can be taken for polyester, including hydrolysis, methanolysis, and glycolysis [14]. Hydrolysis is carried out under neutral, acidic, or basic conditions in an aqueous solution, but the treatment is time-consuming and requires high pressures and temperatures [16]. Methanolysis is based on a methanol solvent, resulting in a mixture of products, which is challenging to separate [17]. Finally, glycolysis is based on a glycol (e.g., ethylene glycol) solvent, resulting in flexible, simple, and mature processes [14]. The three depolymerization pathways are shown in Figure 1.

One of the commercially used recycling methods for polycotton blends, with a higher TRL of 5–6, is alkaline hydrolysis, where the polyester is removed from the fabric in the form of monomers, leaving the cellulose fibers intact [8,18]. The cellulose fibers are then mixed with new forest cellulose and recycled into viscose fibers. An example of this process is Sodra’s OnceMore^®^ fibers, which are turned into clothing. Notably, a collaboration with Lindex resulted in a complete collection for Midsummer 2024 (Midsommer 2024) [19,20].

**Figure 2:**
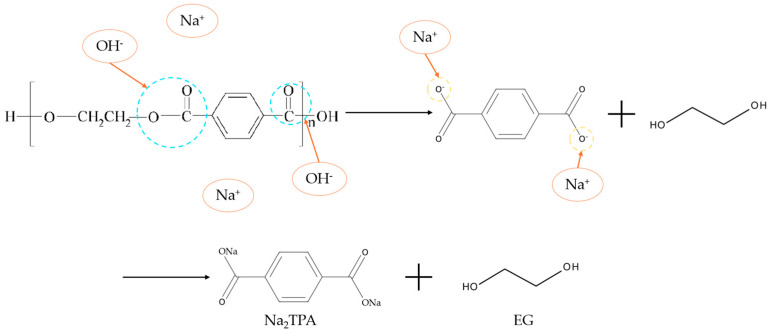
Illustration of the alkaline hydrolysis reaction of PET molecule towards the formation of Na_2_TPA and EG.

Alkaline hydrolysis uses high temperatures of 120–200 °C and high NaOH concentration (5–20% *w*/*v*) to hydrolyze the ester bonds of polyethylene terephthalate (PET), breaking the polymer into its constituents: ethylene glycol (EG) and disodium terephthalate (Na_2_TPA) [16,18,21,22]. A schematic of this reaction is presented in Figure 2. Terephthalic acid (TPA) can be recovered at high purity from Na_2_TPA by treatment with sulfuric acid [16]. However, the high temperatures required for this process increase the energy consumption and may lead to cellulose fiber degradation in polycotton blends. To address these issues, modifying the process to include additional solvents, particularly ethanol, offers a potential solution [22]. This adjustment allows the temperature to be reduced to below 100 °C without negatively affecting PET depolymerization, while also significantly shortening the reaction time. For instance, where conventional alkali hydrolysis of PET at 90 °C typically requires 6–24 h, the alcohol–water alkali process can achieve the same result in less than 1 h [22].

The aim of this paper is to develop a new process based on the alcohol–water alkali method that enables the treatment of textile waste, focusing on reducing energy requirements and minimizing the volumes of chemical waste typically associated with state-of-the-art alkali hydrolysis processes [8,22]. This approach results in a heated high-ethanol alkaline aqueous (HHeAA) process. Additionally, we propose downstream operations capable of reusing reaction chemicals and water in a closed loop, thereby preventing waste generation.

## 2. Materials and Methods

### 2.1. Small-Scale Experiments

For the experiments, lab coats (product number 113-8748; VWR, Radnor, PA, USA) with a composition of 65% polyester and 35% cotton were used. The lab coat fabric was cut into square pieces with dimensions of 4 × 4 cm. In each experiment, 2.4 g of textile was used (1.56 g polyester and 0.84 g cotton). As a control, plain woven 100% cotton (Ohlssons Tyger & Stuvar AB, Luleå, Sweden) was used. In each experiment, 0.84 g of cotton was used.

The reaction solution was a mixture of distilled water, absolute ethanol (≥99.8% *v*/*v*; VWR, Radnor, PA, USA), and sodium hydroxide (Sigma-Aldrich, St. Louis, MO, USA). The ratio of NaOH to PET was maintained at a 2:1 molar ratio, resulting in a NaOH concentration of 1.3% *w*/*v*. For each experiment, 90 mL of absolute ethanol and 10 mL of distilled water were used, resulting in a 90% *v*/*v* EtOH solution. In each experiment, 100 mL of the solution was used.

The pieces of fabric and the solution were placed inside a 250 mL stirred pressure reactor autoclave system (Amar Equipment, Mumbai, India). The mixture was then heated to the set temperature and left to react for the specified duration. After the treatment, the system was cooled to room temperature (~25 °C). The reaction mixture was collected in a beaker and diluted with 167 mL of distilled water, not only to lower the concentrations of NaOH and EtOH, but also to dissolve the solid Na_2_TPA. The reaction mixture was then vacuum-filtered. The filtrate was collected for further analysis, and the pre-weighed filter paper with the treated fabric was washed with 167 mL of distilled water. This second filtrate (wash) was also collected for analysis. Finally, the fabric was washed with additional water to remove any remaining soluble impurities. The filter paper was transferred to an oven at 60 °C to dry overnight. The dry weight was then measured, and the hydrolysis yield percentage was calculated (Equation (1)).

(1) $$PET\;Hydrolysis\;\left(\%\right)=\left(1-\frac{g_{Recovered\;fabric}}{0.65\times 2.4\;g}\right)\times 100$$

### 2.2. Scaled Experiments and Fractionation

**Figure 3:**
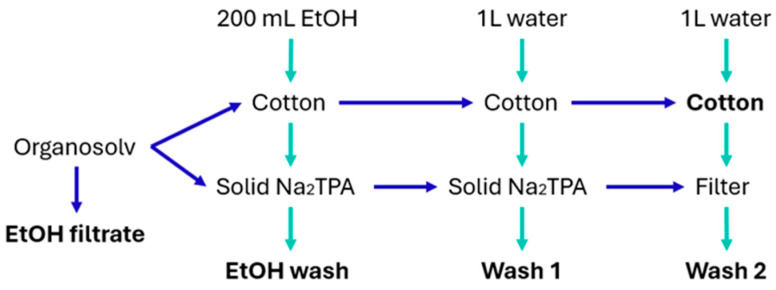
Fractionation process of the scaled process.

The scaled experiment was carried out with 24 g of lab coat fabric samples that were cut into approximately 1 × 4 cm strips, with one piece of 4 × 4 cm for further analysis. The fabric was mixed with 1 L of 90% *v*/*v* ethanol aqueous solution containing 12.97 g of NaOH, to provide a 2:1 NaOH-to-PET ratio. The mixture was placed in an air-heated multi-digester system with 6 × 2.5 L batch autoclave reactors, and the treatment was carried out at 90 °C for 20 min. The sample was cooled down and filtered through a 1 mm sieve to separate the fabric and slurry. The slurry was filtered through a cellulose filter (Qualitative 401, VWR, Radnor, PA, USA), resulting in an ethanol-rich filtrate (EtOH filtrate fraction) and a solid Na_2_TPA fraction. Three wash steps followed: (1) ethanol wash with 200 mL to remove EG from the fabric and the solid Na_2_TPA, resulting in the ‘EtOH wash’ fraction; (2) distilled water wash of 1 L to dissolve the Na_2_TPA crystals from the fabric and the filtration cake, resulting in a Na_2_TPA-rich fraction called ‘Wash 1’; and (3) distilled water wash of 1 L to wash any leftover Na_2_TPA from the fabric and the filtration system, resulting in ‘Wash 2’ (Figure 3). The recovered cotton fabric was dried, weighed, and stored at room temperature in plastic bags. The four liquid fractions were stored in plastic bottles at room temperature.

Finally, trials to decrease the liquid-to-solid ratio (LSR) were conducted. The solid loading was increased from 24 g to 48, 72, 96, 120, and 144 g, corresponding to a decrease in LSR from 42 to 21, 14, 10, 8, and 7. The 2:1 NaOH-to-PET ratio was maintained for all trials. The fractionation and washes were performed as described above.

### 2.3. Mass Balances

Mass balances were carried out for the scaled experiments to provide insights into the recovery of EG, TPA, NaOH, and cotton in all recovered fractions. The EG content was determined directly from the liquid fractions using HPLC (see Section 2.4.5). The EtOH filtrate and EtOH wash were evaporated (Heidolph, Schwabach, Germany), and the leftover was dissolved in 100–150 mL of distilled water. Subsequently, all aqueous samples were processed in the same manner. The whole volumes of the aqueous solution of EtOH filtrate and EtOH wash, as well as Wash 1 and Wash 2, were acidified to a pH of 2 by using 1 M H_2_SO_4_, resulting in the conversion of Na_2_TPA to TPA (which was precipitated) and Na_2_SO_4_. The precipitate was filtered out and dried overnight in an oven at 50 °C. The filtrate (containing the Na_2_SO_4_) was neutralized with NaOH, evaporated in a rotary evaporator, and kept in an oven at 90 °C before being weighed. The Na_2_SO_4_ was further washed with ethanol to extract any leftover EG, followed by filtration to recover the solid Na_2_SO_4_. The TPA and Na_2_SO_4_ fractions were then analyzed for their purity (see Section 2.4).

### 2.4. Analytics

#### 2.4.1. Scanning Electron Microscopy

The morphology of the fibers was observed by scanning electron microscopy (SEM) using an FEI Magellan 400 field-emission XHR-SEM (Thermo Fisher Scientific™, Waltham, MA, USA). The samples were placed on conductive carbon tape prior to the analysis, and the images were taken at a low accelerating voltage of 2 kV and a beam current of 3.1 pA.

#### 2.4.2. Degree of Depolymerization

The degree of polymerization (DP) was determined using the ISO 5351:2010 method through the determination of limiting viscosity number in cupri-ethylenediamine solution [23]. Only pure cotton samples were used to study the effect of the treatment on the DP value. An untreated sample was compared to samples treated at 90 °C for 20 min, at 80 °C for 60 min, and at 70 °C for 105 min.

#### 2.4.3. Fourier-Transform Infrared Spectroscopy

The spectra of untreated and treated textiles were measured using Nicolet™ Summit FTIR (Thermo Fisher Scientific™, Waltham, MA, USA) with an Everest ATR and diamond crystal plate. The wavelength measurement range was fixed at 400–4000 cm^−1^ with a resolution of 4 cm^−1^ and 16 scans.

#### 2.4.4. Nuclear Magnetic Resonance

Nuclear magnetic resonance (NMR) spectroscopy was performed on a 60 MHz X-pulse Benchtop NMR spectrometer (Oxford Instruments, Abingdon-on-Thames, UK). The purity of the produced TPA was checked by ^1^H NMR measurements. In brief, ~20 mg of TPA was dissolved in 6 μL DMSO-d_6_ (Cambridge Isotope Laboratories, Tewksbury, MA, USA). The solution was transferred to a standard 5 mm NMR tube and placed into the tabletop NMR. The purity was determined by observing the presence of the peak corresponding to EG (3.5–4 ppm) in the ^1^H NMR spectrum, which is considered to be a contaminant.

#### 2.4.5. High-Performance Liquid Chromatography

EG was analyzed by high-performance liquid chromatography (HPLC) on an apparatus equipped with a refractive index detector (PerkinElmer, Waltham, MA, USA) and (BioRad, Hercules, CA, USA) operated at 65 °C with 5 mM H_2_SO_4_ as the mobile phase and a flow rate of 0.6 mL/min. All samples of EG were acidified to pH 2 and filtered through hydrophilic 0.2 μm filters to remove any Na_2_TPA, which could cause damage to the system.

#### 2.4.6. Ash Analysis

Inorganic ash was determined gravimetrically by ashing samples of TPA and Na_2_SO_4_ at 550 °C for 3 h.

#### 2.4.7. Yarn Tensile Properties

The untreated yarns were extracted directly from the fabric of the lab coat and the reference cotton fabric in both directions. The samples were conditioned in a standard atmosphere according to ISO 139:2005 [24]. The tensile properties were measured according to ISO 2062 using MesdanLab Strength Tester (Mesdan, Italy), with a gauge length of 250 mm, clamp speed of 250 mm·min^−1^, load cell of 0.1 KN, and pretension of 10 cN [25]. Yarns after the chemical treatment underwent testing using the FAVIMAT+ instrument (TexTechno, Mönchengladbach, Germany) tester with a load cell of 210 cN, test speed of 10 mm·min^−1^, and pretension of 0.01 cN/Tex. Five yarns were collected from each sample after treatment and were subjected to the standard conditions prior to measurements.

## 3. Results

### 3.1. Small-Scale Pretreatment

**Figure 4:**
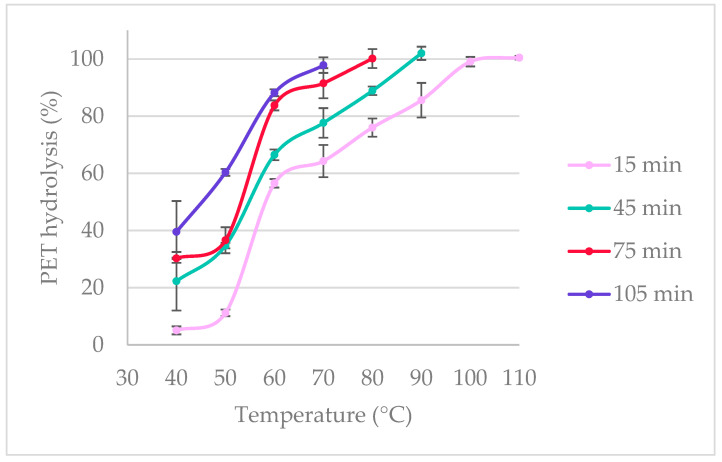
PET hydrolysis yields at retention times of 15, 45, 75, and 105 min at temperatures from 40 °C to 110 °C.

**Figure 5:**
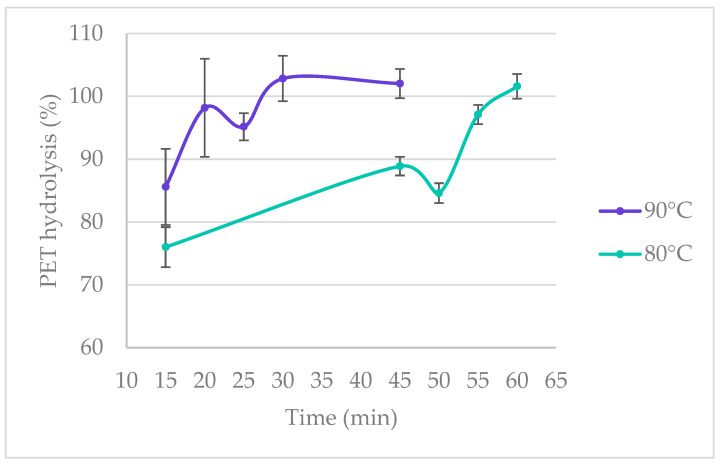
PET hydrolysis yields at 80 °C and 90 °C under different retention times.

The initial study was carried out with four retention times of 15, 45, 75, and 105 min (Table 1). The starting temperature was 40 °C, which was increased by 10 °C increments until 97% hydrolysis of PET was reached. At 40 °C, low hydrolysis yields were observed; 5.1% was obtained at 15 min, and 39.5% was obtained at 105 min (Figure 4). At 60 °C, the PET hydrolysis yields improved significantly at all retention times, with 56.5% obtained at 15 min and 88.2% at 105 min. Complete PET hydrolysis was obtained at 100 °C for 15 min, 90 °C for 45 min, 80 °C for 75 min, and 70 °C for 105 min. For an industrial application, it would be ideal to maintain the retention time low and the temperature below 100 °C to avoid the need for high-pressure equipment. For this reason, the treatment time for the temperatures of 80 °C and 90 °C was gradually decreased. It was found that at 90 °C, the retention time can be shortened by half to achieve a hydrolysis yield of over 95%. After 20 min of treatment, 98.1% of PET hydrolysis was achieved (Figure 5). For the temperature of 80 °C, the retention time could be lowered from 75 min to 60 min, where complete hydrolysis occurs, or to 55 min where 97.1% was achieved (Table 2).

**Figure 6:**
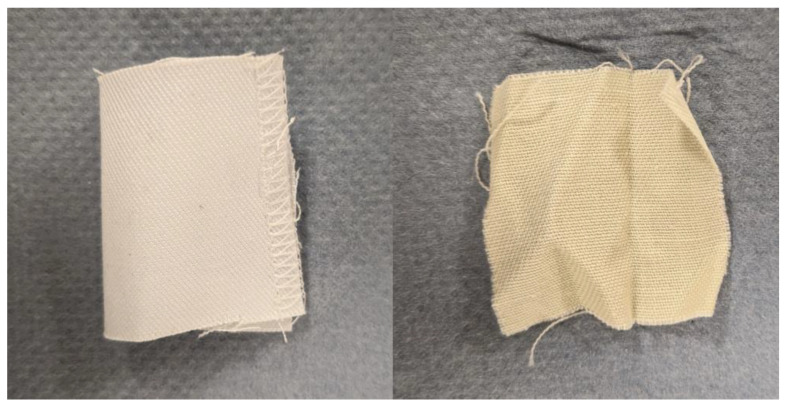
Photograph of untreated lab coat piece (**left**) and lab coat piece treated at 90 °C for 20 min (**right**). The removal of PET is evident by the loosened structure of the fabric.

Pure cotton samples were treated at 90 °C for 20 min, at 80 °C for 60 min, and at 70 °C for 105 min to study the effect of the process on the cotton fiber. The loss in the initial weight was 11.6%, 11.5%, and 8.2% for 90 °C, 80 °C, and 70 °C, respectively. The color of the textile changed from white to slightly beige (Figure 6), suggesting removal of initial sizing, finish, or coating. However, the hydrolysis of the cotton sample was very low, showing that the HHeAA process does not affect the cotton fiber.

The current chemical recycling technologies provide the option to hydrolyze PET through alkali hydrolysis. However, it is important to point out the crucial aspects where the present study improves on the current technology. First, using water as a sole solvent can lead to complete hydrolysis, but the required conditions can be rather harsh. For example, a hydrolysis yield of 98% requires treatment at 200 °C for 1 h. For a reaction temperature of 120 °C, the maximum yield achieved was only 33% after 7 h with 45 g/L NaOH [16]. Furthermore, for a 30 min reaction and a concentration of 15 wt% of NaOH, the yield achieved was close to 50% at 80 °C, while for 5 wt% NaOH, at 90 °C and 1 h reaction time, the yield reached almost 70% [26]. Another solution for achieving higher hydrolysis rates at lower temperatures and shorter times is to add catalysts to the reaction mixture. The study conducted by Barredo et al. (2023) showed that at 90 °C for 4 h, the PET conversion without using a catalyst was only 20%, while with the addition of a catalyst, it reached up to 90%. The NaOH concentration used in this study was ~70 g/L [27]. It is clear that by using either lower NaOH concentrations or lower temperatures, high yields cannot be achieved.

However, adding ethanol to the reaction mixture significantly reduces the time and temperatures required for PET hydrolysis, as ethanol can facilitate the reaction between sodium hydroxide ions, which are dissolved in water, and the hydrophobic PET [22]. According to the study, for a solution of 90 vol% EtOH, complete hydrolysis was achieved at 80 °C after 90 min using 0.2 g/mL KOH [22]. However, this concentration (200 g/L) is very high and can become challenging for a possible scaled procedure. By using less KOH (50 g/L), a yield of 99.4% was achieved at 80 °C, but the time rose to 180 min. Another study showed that the best results were achieved by using 60 vol% EtOH and 5 wt% NaOH at 80 °C for 20 min, which led to a 95% yield. The rest of the results were below 80% [21].

With the current study, it has become clear that nearly complete hydrolysis of PET could be achieved in various combinations of time and temperature, such as at 90 °C for 20 min and 12.9 g/L of NaOH in 90 *v*/*v*% of EtOH solution. This is a significant improvement because by using such low temperatures and treatment times, as well as reduced alkali concentration, the cotton fibers could retain their properties, making them more suitable as raw material for new textiles. The NaOH concentration used in the current study is significantly lower than what is commonly used in the literature, making it safer to handle and reducing the need for chemicals. Additionally, a short reaction time was achieved, which can be beneficial for the future continuous operation of the process.

### 3.2. Analytics

Based on the above, the following samples were chosen for further analysis: the lab coat treated at 90 °C for 20 min, at 80 °C for 60 min, and at 70 °C for 105 min. Cotton was also treated at these conditions to determine the direct effect of the treatments on the cotton fibers.

**Figure 7:**
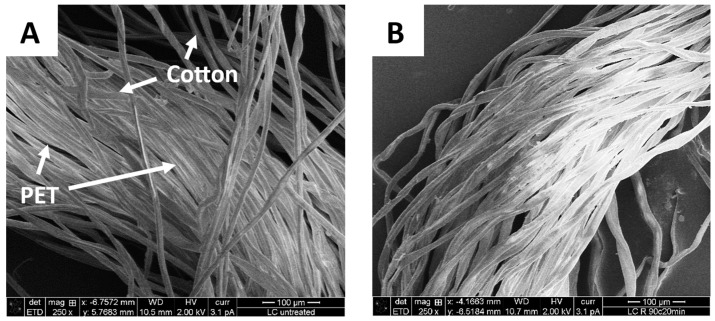
SEM image showing the composition of the initial polycotton fiber (**A**), containing PET and cotton fibers, and the PET removal from the lab coat fibers after the treatment at 90 °C for 20 min (**B**).

**Figure 8:**
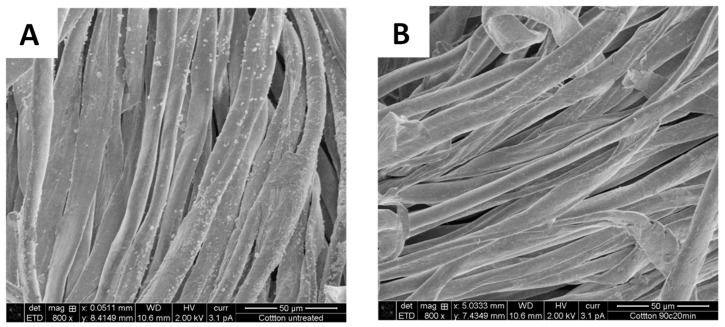
SEM image of pure cotton sample before (**A**) and after (**B**) treatment at 90 °C for 20 min.

Scanning electron microscopy imaging was performed to observe the removal of PET fibers from the polycotton yarn, as well as to provide a visual observation of the fiber quality and potential changes in their structure. Figure 7 shows the initial polycotton yarn consisting of PET fibers and cotton fibers, where the PET fibers are smooth, well defined, and oriented in one direction, whereas the cotton fibers appear as convoluted ribbons within the yarn [28]. After the treatment at 90 °C for 20 min, only cotton fibers are present in the sample, which confirms the complete removal of PET from the polycotton yarn. Similar results were observed by Andini et al. when the treatment of the polycotton sample led to the removal of PET fibers, leaving the cotton fiber mostly intact [28]. As an additional control, a sample of pure cotton was treated at 90 °C for 20 min, and the obtained yarn was compared to the untreated cotton yarn. Figure 8 shows no significant changes in the fiber structure. However, the small particles that were present on the surface of the initial fibers were removed during the treatment, which would explain the observed minor hydrolysis in the pure cotton trials. The particles could be the initial sizing, finish, or coating.

**Figure 9:**
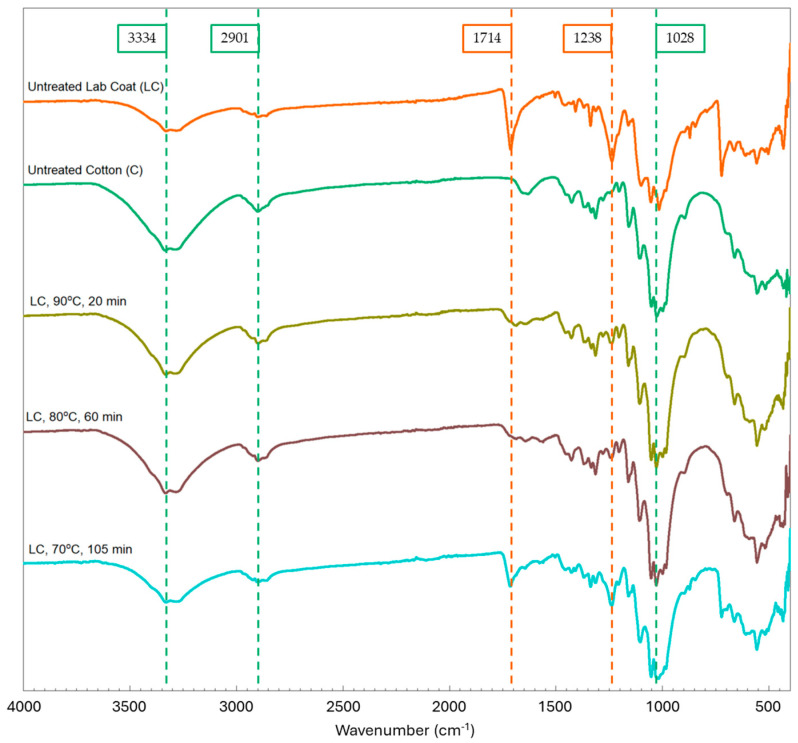
FTIR spectra of cotton and lab coat controls and lab coat after the treatment with characteristic PET peaks at 1714 and 1238 cm^−1^.

**Figure 10:**
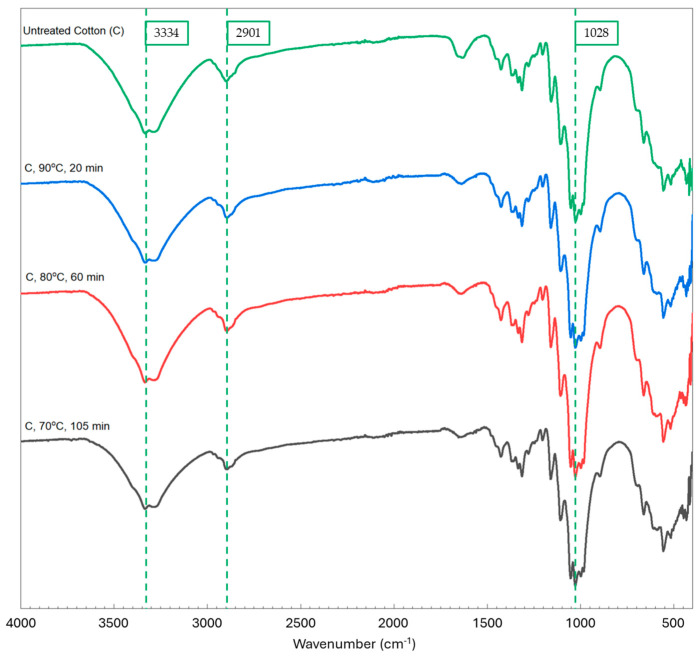
FTIR spectra of pure cotton sample before and after treatment at 90 °C for 20 min, 80 °C for 60 min, and 70 °C for 105 min showing the characteristic peaks for cotton at 3334, 2901, and 1028 cm^−1^.

The FTIR spectra in Figure 9 show the untreated controls of lab coat fabric and pure cotton, as well as all the pretreated lab coat samples (90 °C, 20 min). The untreated lab coat has the characteristic peaks of both cotton (3334, 2901, and 1028 cm^−1^) and PET (1714 and 1238 cm^−1^) [29,30], whereas all pretreated lab coat samples display only the characteristic peaks for cotton, except for the sample treated at 70 °C for 105 min, where a small amount of PET was expected and the characteristic peaks for PET were found. The pure cotton sample was also subjected to FTIR analysis, and no changes were observed in the spectra of the treated samples (Figure 10). The peaks from the untreated samples of cotton and lab coat, alongside the treated lab coat at 90 °C for 20 min, were correlated with structural information and are shown in Table 3 [31,32,33]. It is also interesting to note that in the treated sample, more peaks correlated to cotton appear as the sample is stripped of the polyester after treatment.

In a treatment like HHeAA, due to the alkali conditions and elevated temperatures, depolymerization of cotton can take place. Under these conditions, NaOH ions can attack and break some hydrogen bonds in cellulose molecules (the main component of cotton). However, this reaction also depends on the treatment time [34,35,36]. The degree of polymerization was determined to study the effect of the treatment conditions on the cotton fiber quality (Table 4). The DP value of 1584 was obtained for the untreated cotton. With increasing time of treatment, the DP value decreased in the cotton sample, even if the temperature was higher in the shorter time of treatment. Cotton treated at 90 °C for 20 min yielded a DP value of 1111, and that treated at 80 °C for 60 min resulted in a DP value of 916. Finally, the DP value in the cotton sample treated at 70 °C for 105 min was 720. The DP value determines the potential for future use of the recovered fibers. Cotton fibers with a DP of around 1000 and higher can be used for the production of regenerated fibers, whereas fibers with lower DP can only be used for other applications such as the production of cellulose nanocrystals (DP around 250) and generation of bioenergy (DP around 5) [28]. Thus, the cotton fibers recovered after the treatment at 90 °C for 20 min would be suitable for the production of regenerated fibers such as lyocell or viscose [28,37].

Another evaluation of the cotton yarn left from the treated polycotton blend is presented in Table 5, which shows the results of the tensile strength and elongation at break for both untreated and treated yarns from the lab coat and the cotton reference. The major loss observed was in the tensile strength of the treated lab coat yarns for all the reported temperatures, when compared to the untreated yarns. For the reference cotton samples, the tensile strength was not affected by the treatment; on the contrary, the results indicate a slight but insignificant increase. The major loss of strength can be attributed to the removal of PET material after the chemical treatment from the heterogeneous yarns. PET fibers make up 65% of the yarn’s composition; after their hydrolysis, the integrity and structure of the yarn are significantly compromised with gaps between the remaining fibers, forming weak points along the yarns. This change in the twist factor makes them more susceptible to rupture. Meanwhile, for the pure cotton yarns, the maintenance of the tensile properties indicates that the chemical treatment is compatible with cellulosic materials. Since the treatment was conducted without any tension applied to the samples, the effect of alkali results in slight shrinkage, causing elongation at break to be maintained or slightly increased [38]. Furthermore, the alkali treatment disrupts hydrogen bonds in the cellulose structure, leading to partial separation of chains and thus lowering the DP in accordance with the obtained results. This leads to the formation of more amorphous regions which may cause slight loosening of the cotton structure and increase the surface texture, resulting in an increase in the elongation at break of the cotton yarns [39].

Therefore, the treatment at 90 °C for 20 min would be preferred, to reduce the time of the treatment while obtaining similar results in regard to the tensile properties of the yarns.

### 3.3. Scaled HHeAA Process

**Figure 11:**
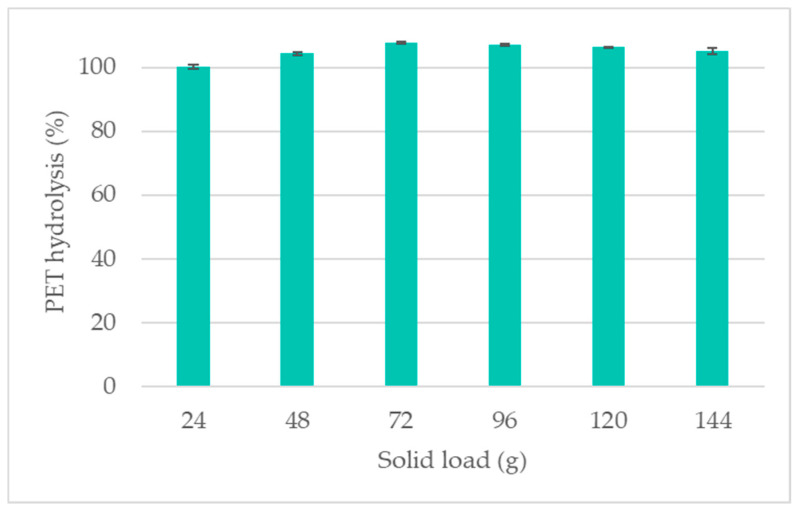
PET hydrolysis in the scaled trials with different solid loadings. The reaction volume was maintained at 1 L.

**Figure 12:**
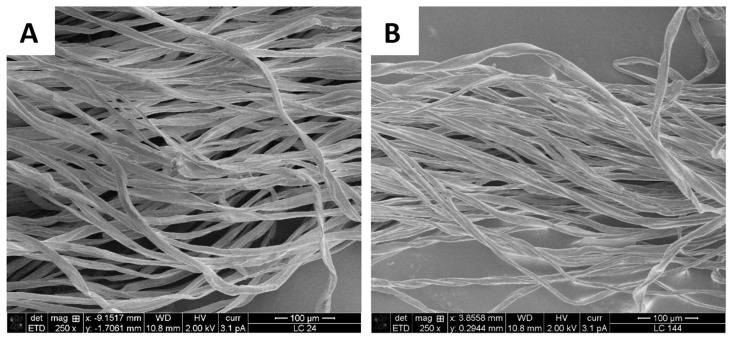
SEM image of lab coat sample pretreated at 24 g (**A**) and 144 g (**B**) of solid load.

**Figure 13:**
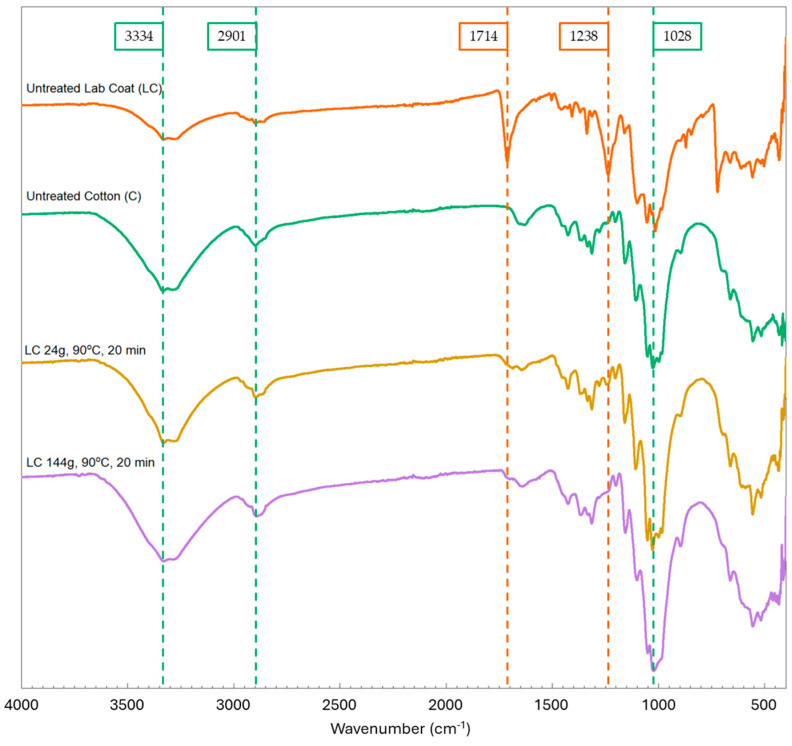
FTIR spectra of lab coat samples treated at the scaled experiment with increasing solid load from 24 g to 144 g.

Based on the small-scale results, scaled trials were carried out. The reaction volume was increased by 10-fold, corresponding to an increase in lab coat weight from 2.4 g to 24 g. Under the selected conditions of 90 °C for 20 min, complete hydrolysis of PET was achieved (Figure 11). This was confirmed through SEM imaging (Figure 12) and FTIR analysis (Figure 13). The additional loss in sample weight could be attributed to the removal of initial sizing, finish, or coating that might be present in the fabric, as was observed in the pretreatment of pure cotton samples on a small scale. In the next step, we aimed to decrease the LSR, which is a crucial factor for process economics [21]. As such, the liquid-to-solid ratio was decreased from 42 to 7 in six steps with corresponding lab coat solid loading of 24, 48, 72, 96, 120, and 144 g. For all the samples, complete hydrolysis of PET was obtained (Figure 11). These data show a significant improvement compared to the current state of the art, which is performed at high LSR (up to 100) with the lowest reported trials at LSR 10 [8,9,16,22,26,27].

**Figure 14:**
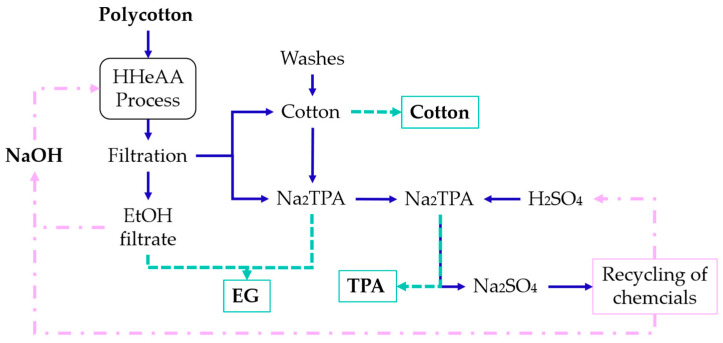
Closed-loop HHeAA process.

Apart from targeting a high PET hydrolysis, it is crucial to develop a proper downstream process with the possibility for recovery of PET monomers and used chemicals when targeting a potential industrial application (Figure 14) [8,9,21]. PET monomers, TPA and EG, must be recovered separately for any further uses, whereas the used chemicals, sodium hydroxide and sulfuric acid, should be recovered so they can be recycled within the treatment system [8,40]. For this purpose, the pretreated slurry was filtered via a 1 mm sieve to separate the textile from the liquid, followed by filtration through a cellulose filter removing the solid Na_2_TPA, resulting in ethanol filtrate. The textile was further washed with ethanol, yielding the ethanol wash. Subsequently, a two-step water wash was performed with 1 L of distilled water. Thus, four fractions were recovered, and each was analyzed for TPA, EG, and NaOH content.

**Figure 15:**
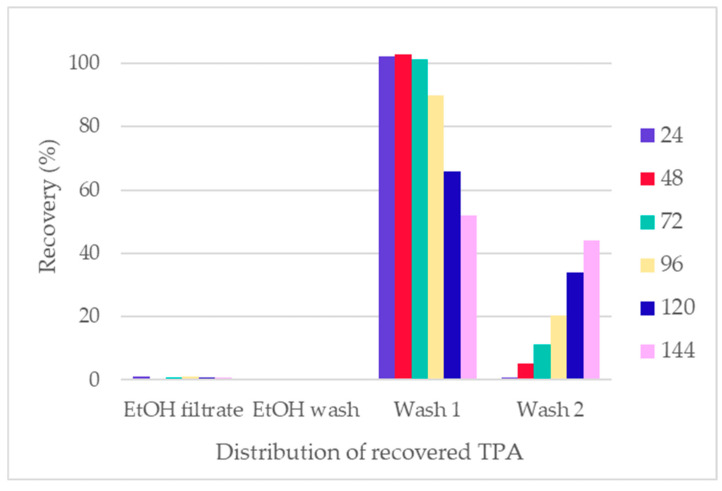
Total recovery of TPA and its distribution within fractions.

**Figure 16:**
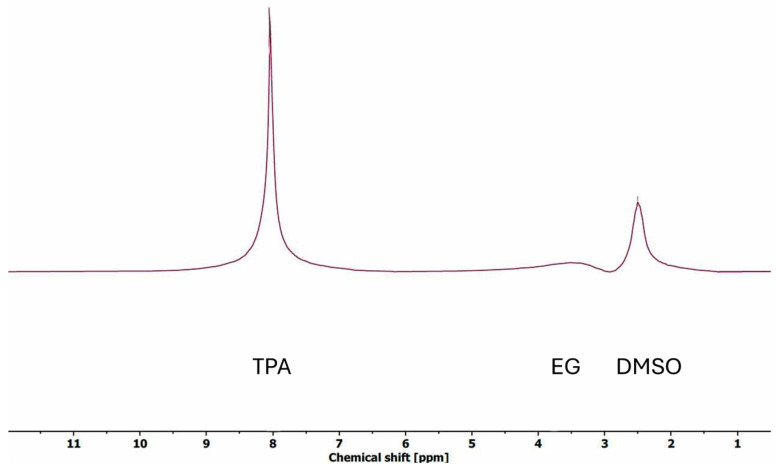
^1^H-NMR spectra of solids recovered as TPA fraction.

The total recovery of TPA in all fractions ranged from 97.1% to 113.4%, suggesting some contamination of the recovered TPA solid with NaOH, EG, and/or the initial sizing, finish, or coating (Figure 15). The NaOH content was accounted for through ash analysis of the samples and subtracted from the presented data. The ash content was up to 5% in Wash 1 of LSR 7. In Wash 2, the highest ash content was 1.6% in the LSR 7 sample. However, additional wash after filtration should remove the excess sodium salt present in the sample. The presence of EG in the TPA solids was analyzed via ^1^H-NMR. The expected peak for TPA is present at 8 ppm, while the EG peak can be located around 3.4 ppm for DMSO-d_6_ solvent [41,42]. Very small amounts of EG were found (10–15%), suggesting that the TPA fraction had a high purity (Figure 16). Thus, the excess recovery of TPA solids could be explained by the presence of contamination from the initial sizing, finish, or coating. The distribution of recovered TPA within the fraction was advantageous for the downstream process as tiny amounts of it were recovered in the EtOH filtrate and EtOH wash fractions (<1.2% and <0.2%, respectively). Na_2_TPA, which results from PET hydrolysis under alkali conditions, is not soluble in the reaction mixture of 90% EtOH with unreacted NaOH concentrations. Depending on the LSR, the concentration of the unreacted NaOH was 6.3–34.0 g/L. Thus, it is easy to separate the solid Na_2_TPA from the EtOH filtrate and EtOH wash. The majority of the Na_2_TPA was recovered in the first water wash as it is soluble in water. With increasing solid load, an increasing amount of Na_2_TPA was recovered in the second water wash.

A study by Rezazadech et al. (2021) shows that the solubility of Na_2_TPA is not considerably affected in the temperature range of 25–70 °C. However, the higher the content of NaOH and EG in the solution, the lower the solubility of the Na_2_TPA. Thus, in water only and at 25 °C, about 13% *w*/*v* of Na_2_TPA should be soluble [43]. The EG and excess NaOH contents in Wash 1 are below 0.04 mol/L and 0.5 mol/L, respectively, which should not cause a significant decrease in the solubility of Na_2_TPA [43]. The highest content of Na_2_TPA in the wash fractions was up to 5% *w*/*v*. Therefore, by improving the efficiency of the wash process, for example, with the addition of press and soak steps, the recovery of Na_2_TPA could be significantly improved in the downstream process, requiring less water.

**Figure 17:**
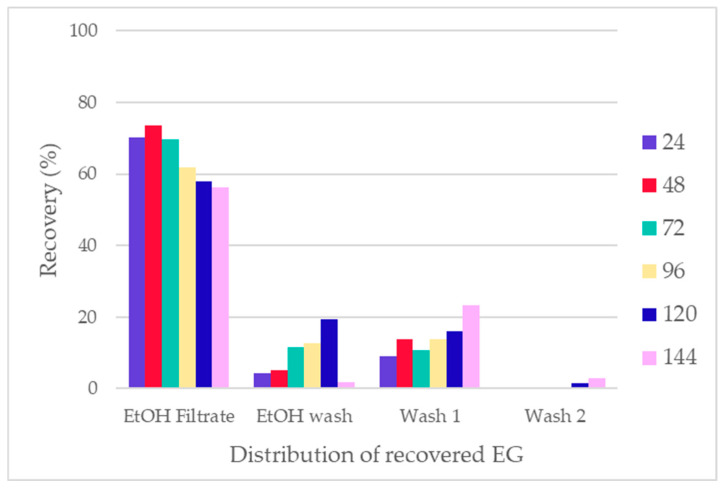
Total recovery of EG and its distribution within fractions.

The total EG recovery after the treatment was 83.9% to 95.1% (Figure 17). The distribution within the fractions showed that 60.9% to 83.4% of EG was recovered in the EtOH filtrate, with 2.0% to 20.5% recovery in the EtOH wash. Generally, less than 20% of total EG was recovered in water washes. This distribution allows for the recovery of EG from the EtOH fractions or the recycling of EG into a new round of treatment, as it was reported that the presence of EG in the treatment can improve the hydrolysis of PET [8,9].

**Figure 18:**
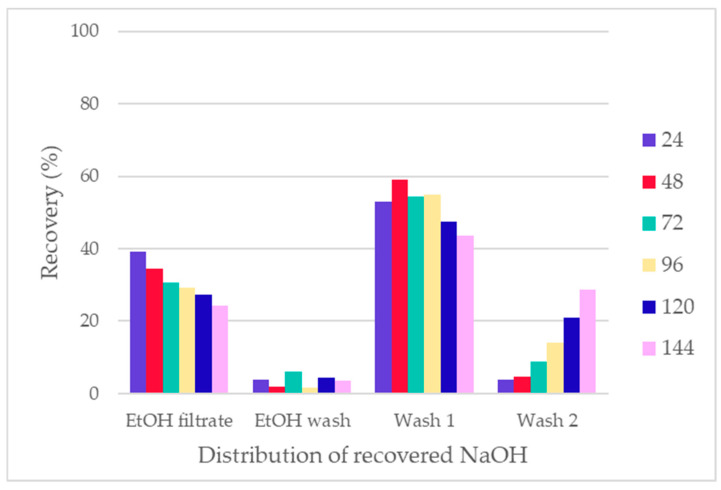
Distribution of recovered NaOH in the form of Na_2_SO_4_.

The NaOH was recovered from the treatment in the form of Na_2_SO_4_ salt, as H_2_SO_4_ was used to precipitate the TPA from the fractions. The recovered Na_2_SO_4_ fractions exhibited high purity (>97%), making them suitable for chemical recycling and recirculation within the treatment system [40]. The distribution of recovered sodium (expressed as NaOH) is shown in Figure 18. The majority of the unreacted NaOH was recovered in the EtOH filtrate, while small amounts were washed out with the EtOH wash. Some of the excess NaOH and the majority of the reacted NaOH were recovered in the water washes, as the distribution of NaOH follows the TPA recovery distribution.

**Figure 19:**
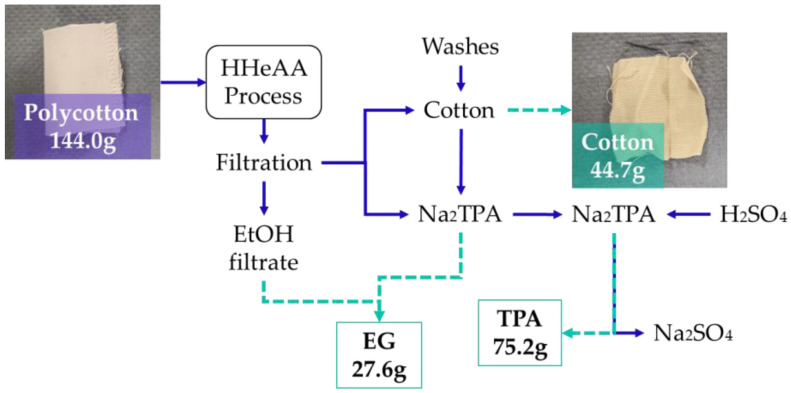
Schematic representation for LSR 7 process and the product outputs.

In Figure 19 and Table 6, the process for LSR 7 is presented schematically. The initial weights are calculated based on the assumption of complete hydrolysis of PET.

## 4. Conclusions

The current study demonstrates a new process for the treatment of polycotton that is capable of separating PET from the cotton fibers at milder conditions and with lower chemical consumption than is typically reported in the literature. The process could be operated successfully at a liquid-to-solid ratio of 7, which is substantially lower than what has been reported, making the HHeAA process more suitable for future industrial implementation.

In addition, FTIR spectra show that cotton fibers recovered at LSR 7 were pure cotton fiber, without PET contamination, which was supported by SEM imaging. The following analysis of the cotton fiber showed a slight decrease in the degree of depolymerization and maintenance of the tensile properties, which indicates that the chemical treatment is compatible with cellulosic materials.

Finally, PET monomers can be recovered separately, allowing their use as building blocks for new plastics or textiles. TPA of high purity was recovered, and the remaining small amount of contamination could be removed with an additional wash. Furthermore, the NaOH and H_2_SO_4_ could be regenerated, leading to a closed-loop procedure. This suggested scenario tackles the two most important challenges to the chemical recycling methods: the high LSR and excessive amounts of water and chemical waste.

## Figures and Tables

**Table 1:** PET hydrolysis yields at retention times of 15, 45, 75, and 105 min at temperatures from 40 °C to 110 °C.

		Hydrolysis (%)			
	Time (min)	15	45	75	105
Temperature (°C)					
40		5.06 ± 1.39	22.26 ± 10.25	30.28 ± 0.27	39.52 ± 10.81
50		11.19 ± 1.14	34.73 ± 0.96	36.60 ± 4.55	60.36 ± 1.21
60		56.52 ± 1.49	66.50 ± 1.88	83.78 ± 1.75	88.21 ± 1.20
70		64.30 ± 5.62	77.66 ± 5.18	91.55 ± 5.26	97.88 ± 2.75
80		76.01 ± 3.18	88.89 ± 1.48	100.18 ± 3.34	
90		85.59 ± 6.05	102.03 ± 2.32		
100		99.09 ± 1.67			
110		100.47 ± 0.62			

**Table 2:** PET hydrolysis yields at 80 °C and 90 °C under different retention times.

		Hydrolysis (%)	
	Temperature (°C)	90	80
Time (min)			
15		85.59 ± 6.05	76.01 ± 3.18
20		98.16 ± 7.82	
25		95.16 ± 2.16	
30		102.84 ± 3.60	
45		102.03 ± 2.32	88.89 ± 1.48
50			84.61 ± 1.58
55			97.10 ± 1.54
60			101.60 ± 1.96

**Table 3:** IR assignments of the main vibrations in the FTIR spectra of untreated cotton, untreated lab coat, and lab coat treated at 90 °C for 20 min.

Wavenumber (cm^−1^)			
Untreated Cotton	Untreated Lab Coat	Lab Coat, 90 °C, 20 min	Assignment
3334	3332	3333	Intra-molecular hydrogen bonding C(3)OH···O(5) C(6)O···(O)H
2901	2901	2900	CH_2_ asymmetrical stretching
	1714		Aromatic ester (C=O) stretch
1428		1429	CH_2_ scissoring
1370	1370	1370	C-H bending
1316		1315	CH_2_ rocking
	1238		Aromatic ester (C-C-O)
1159	1162	1161	Anti-symmetrical bridge C-O-C stretching
1028		1030	C-O stretch
897		899	β-linkage of cellulose
	872		Aromatic (C-H) bond
	723		Aromatic (C-H) bond
662		662	OH out-of-plane bending

**Table 4:** Degree of polymerization of cotton samples.

	Viscosity DP (mL/g)
Untreated Cotton	1584
Cotton, 90 °C, 20 min	1111
Cotton, 80 °C, 60 min	916
Cotton, 70 °C, 105 min	720

**Table 5:** Force and elongation at break of lab coat and cotton samples before and after treatment.

	Lab Coat (PET + Cotton)				Cotton			
		90 °C	80 °C	70 °C		90 °C	80 °C	70 °C
	Untreated	20 min	60 min	105 min	Untreated	20 min	60 min	105 min
F_max_ (cN)	913 ± 160	86 ± 13	63 ± 10	85 ± 9	314 ± 48	362 ± 73	396 ± 61	367 ± 96
E_max_ (%)	22 ± 2	9 ± 1	9 ± 1	9 ± 1	13 ± 1	16 ± 2,5	15 ± 2	14 ± 2

**Table 6:** Initial and recovered weights of the products for LSR 7, and their recovery %.

	Initial Amount (g)	Recovered Amount (g)	% of Initial
Cotton	50.4	44.7	88.7
TPA	75.5	75.2	99.6
EG	32.8	27.6	84.1

## Data Availability

The original contributions presented in the study are included in the article, further inquiries can be directed to the corresponding author.
